# Elevated red blood cell distribution width predicts poor prognosis in hilar cholangiocarcinoma

**DOI:** 10.18632/oncotarget.22694

**Published:** 2017-11-25

**Authors:** Bei Li, Zhen You, Xian-Ze Xiong, Yong Zhou, Si-Jia Wu, Rong-Xing Zhou, Jiong Lu, Nan-Sheng Cheng

**Affiliations:** ^1^ Department of Biliary Surgery, West China Hospital of Sichuan University, Chengdu 610041, Sichuan Province, China

**Keywords:** hilar cholangiocarcinoma, red blood cell distribution width, systemic inflammatory response, prognosis

## Abstract

**Background:**

Although the red blood cell distribution width (RDW) has been reported as a reliable predictor of prognosis in several types of cancer, the prognostic value of RDW in hilar cholangiocarcinoma (HC) has not been studied.

**Methods:**

A retrospective analysis of 292 consecutively recruited HC patients undergoing radical resection was conducted. The optimal cutoff value of RDW was determined by the receiver operating characteristic curve (ROC). Survival analysis by the Kaplan-Meier method, the difference between the clinico-pathologic variables and survival were evaluated by log-rank analysis. Multivariate analysis identified independent prognostic risk factors of overall survival (OS).

**Results:**

ROC analysis suggested that the optimal cutoff value for the RDW was 14.95. Linear correlation analysis revealed that RDW is associated with white blood cell count (P = 0.007), neutrophil-to-lymphocyte ratio (P = 0.02), and hemoglobin (P < 0.001), albumin (P < 0.001). In a multivariate analysis, the RDW was an independent prognostic factor for OS (HR = 1.755, 95% CI 1.311-2.349, P < 0.001).

**Conclusions:**

Elevated RDW may be regarded as an indicator of systemic inflammatory response which might facilitate HC growth and metastasis. Current evidence suggests that RDW may have clinical significance in predicting OS after surgery in HC patients.

## INTRODUCTION

Hilar cholangiocarcinoma (HC) is a neoplasm arising from the biliary epithelium at the common hepatic duct bifurcation, and may extend to intrahepatic biliary tree and liver [1, 2]. Primary sclerosing cholangitis, hepatolithiasis, biliary parasitic disease, hepatitis, choledochal cysts, and thorotrast exposure have been identified as risk factors associated with the development of HC [3, 4]. Despite advances in surgical techniques and instruments, the prognosis of HC patients remains extremely poor, with a 5-year overall survival (OS) rate of 10–44% [5–7].

Red blood cell distribution width (RDW) is a routine laboratory parameter examined with the complete blood count test that indicates the heterogeneity in the size of circulating erythrocytes. RDW is also a widely used laboratory parameter for inflammatory diseases [8]. Recent studies showed that RDW is correlated with prognosis in several malignances, such as lung cancer [9], prostate cancer [10] esophageal cancer [11] and hepatocellular carcinoma [12]. Beyazit Y et al reported that RDW can be considered as a supportive diagnostic tool in differentiating between benign and malignant causes of obstructive jaundice [13]. However, to our knowledge, no studies regarding the prognostic value of RDW in HC are available.

The purpose of the present study was to investigate the prognostic value of the preoperative RDW in HC patients.

## RESULTS

The characteristics of the patients are outlined in Table 1. The 292 enrolled patients, including 161 men and 131 women with a median age of 60 years (20–78 years), underwent radical resection for HC. Pre-operative biliary drainage was performed in 194 (81.9%) of the 237 obstructive jaundice patients, 136 patients underwent percutaneous transhepatic cholangiodrainage (PCTD) and 58 patients underwent endoscopic retrograde cholangiopancreatography (ENBD). Preoperative portal vein embolization was performed in 13 patients.

**Table 1:** Baseline characteristics of patients with hilar cholangiocarcinoma according to red blood cell distribution width (RDW) levels

Characteristics	Total	RDW<14.95	RDW>14.95	P value
Gender				0.438
Male	161	75	86	
Female	131	67	64	
Age				0.974
<65	210	102	108	
>65	82	40	42	
Bismuth-Corlette classification				0.567
I	29	12	17	
II	35	16	19	
III	120	56	64	
IV	108	58	50	
Differentiation				0.154
Well/moderate	226	115	111	
Poor	66	27	39	
T stage				0.168
T1	10	8	2	
T2	216	106	110	
T3	60	25	35	
T4	6	3	3	
N stage				0.001
N0	196	104	82	
N1	106	38	68	
AJCC stage				0.001
I	11	9	2	
II	127	74	53	
III	145	55	90	
IV	9	4	5	
Perineural invasion				0.616
Present	136	64	72	
Absent	156	78	78	
Portal vein invasion				0.252
Present	53	22	31	
Absent	239	120	119	
Hepatic artery invasion				
Present	22	8	14	0.231
Absent	270	134	136	
Tumor size				0.066
<30mm	191	100	91	
>30mm	100	41	59	
Transfusion				0.586
Yes	124	58	66	
No	168	84	84	

According to the Bismuth-Corlette classification system, 29 patients (9.9%) were staged as type I, 35 (12.0%) patients as type II, 120 (41.1%) patients as type III and 108 (37.0%) patients as type IV. The radical surgery procedures included extrahepatic bile duct resection (n=22, 7.5%), extrahepatic bile duct resection combined with hepatectomy (n=270, 92.5%, 139 left hemihepatectomy, 3 left trisegmentectomy, 26 mesohepatectomy, 59 right hemihepatectomy, 12 right trisegmentectomy, and 31 caudate lobectomy). Caudate lobectomy was conventionally performed, except for 22 type I patients with sufficient negative margins. The average operative time was 390.8±123.9 min and the median blood loss volume was 500 ml (100–3000 ml). A total of 124 patients underwent intraoperative transfusions.

**Figure 1:**
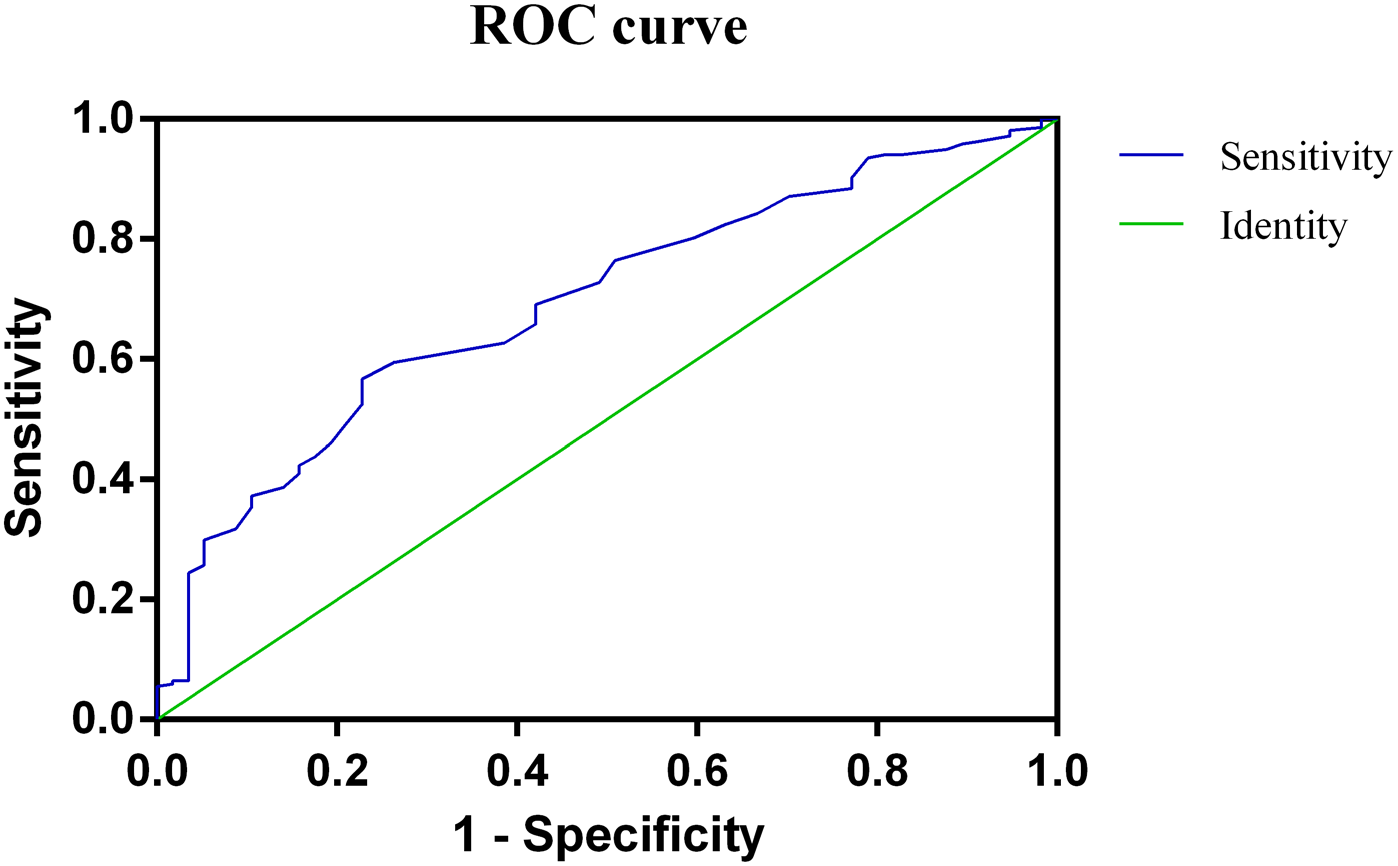
Optimized cutoff value was determined for red blood cell distribution width (RDW) using receiver operating characteristic curve (ROC) analysis

The ROC curve analysis suggested that the optimal cutoff value for the RDW was 14.95 (Figure 1). It indicated that RDW predicts HC prognosis with a sensitivity of 57.6% and a specificity of 77.2 % (AUC = 0.694, 95% CI: 0.621-0.766, P < 0.001). Of the total of 292 patients, 142 patients (48.6%) were detected with RDW of less than 14.95, while there were 150 patients (51.4%) whose RDW was greater than 14.95.

**Figure 2:**
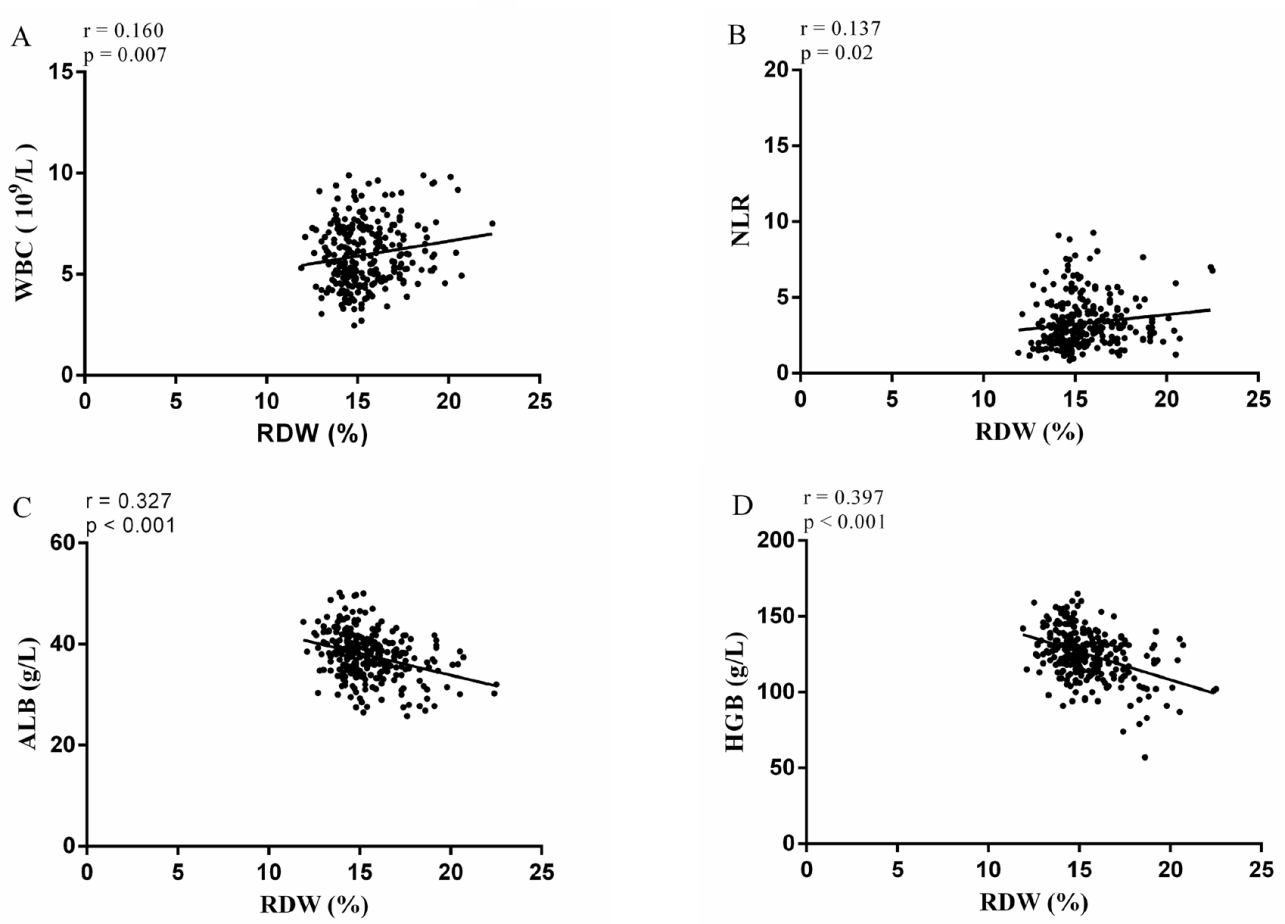
Correlation analysis of RDW with **(A)** WBC, **(B)** NLR, **(C)** ALB, **(D)** HGB. WBC = white blood cell count, NLR = neutrophil-to-lymphocyte ratio, ALB = albumin, HGB = hemoglobin, RDW = red blood cell distribution width.

The relationships between RDW and clinical characteristics are shown in Tables 1 and 2. Our study indicated that N stage, AJCC stage, albumin (ALB), hemoglobin (HGB), white blood cell (WBC), neutrophil-to-lymphocyte ratio (NLR) and mean corpuscular volume (MCV), in the two groups, show significant differences. There were no significant differences in gender, age, Bismuth-Corlette classification, differentiation, T stage, perineural invasion, portal vein invasion, hepatic artery invasion, tumor size, transfusion, platelet count or operation time between the two groups. Linear correlation analysis revealed that RDW > 14.95 is associated with higher WBC count, higher NLR, lower ALB and lower HGB (Figure 2).

**Table 2:** Baseline characteristics of patients with hilar cholangiocarcinoma according to red blood cell distribution width (RDW) levels

Variables	PDW < 16.55	PDW > 16.55	P value
Age (year)	60 (20-78)	59 (20-76)	0.585
ALB (g/L)	38.8 ± 5.0	36.4 ± 4.7	< 0.001
HGB (g/L)	131.0 ± 14.6	121.5 ± 12.3	< 0.001
WBC (×10^9^/L)	5.6 (3.47-9.49)	6.16 (3.7-9.90)	0.035
NLR	2.61 (0.86-17.54)	3.12 (1.21-17.02)	< 0.001
PLT (×10^9^/L)	204.7 ± 74.1	220.0 ± 78.8	0.086
MCV (%)	93.6 (67.4-107)	92.8 (64.5-104.7)	0.039
Operation time (min)	392.2 ± 119.3	389.5 ± 128.6	0.855

Data are expressed as means (SD) or median (IQR). ALB, albumin; HGB, hemoglobin; WBC, white blood cell count; NLR, neutrophil-to-lymphocyte ratio; PLT, platelet count; MCV, mean corpuscular volume.

**Figure 3:**
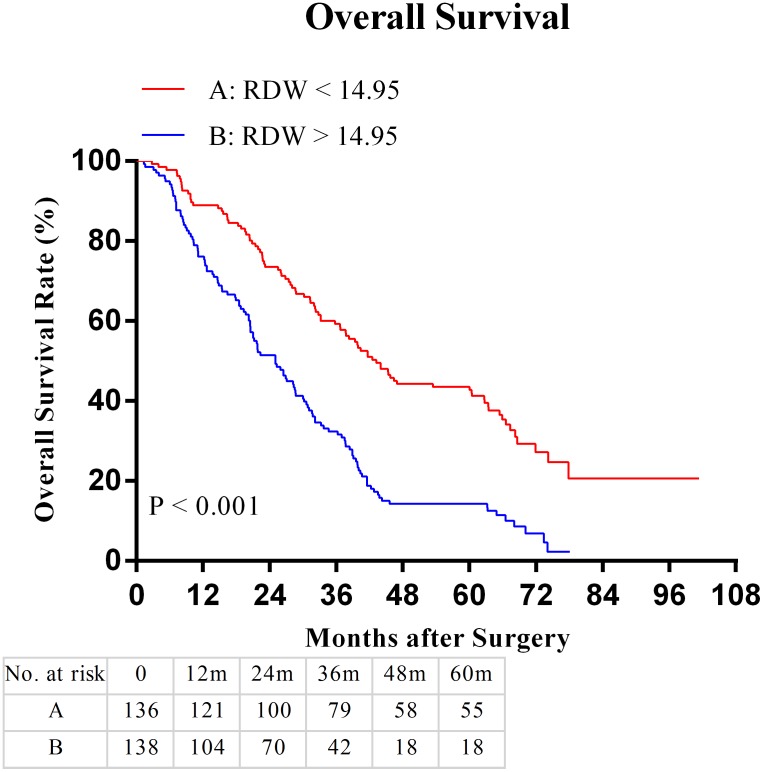
Overall survival (OS) based on red blood cell distribution width (RDW) in hilar cholangiocarcinoma patients with radical resection: the 5-year OS rate in the RDW < 14.95 group were significantly higher than the RDW >14.95 group

In this cohort, the 5-year OS was 25%. Patients with RDW > 14.95 had a significantly worse 5-year OS than patients with RDW < 14.95 (12.0% vs. 38.7%, P < 0.001). The Kaplan-Meier OS curves showed a significant separation in the two subgroups (Figure 3).

Univariate and multivariate analysis were performed and the results were presented in Table 3. Univariate analysis showed that RDW (P <0.001), differentiation (P < 0.001), T stage (P < 0.001), N stage (P < 0.001), AJCC stage (P < 0.001), portal vein invasion (P < 0.001) and hepatic artery invasion (P = 0.002) significantly influenced OS. All factors with P < 0.05 in the univariate analysis were included in the Cox regression model, in which RDW (P < 0.001), differentiation (P = 0.004), N stage (P < 0.001) and AJCC stage (P =0.023) were independent prognostic risk factors for OS.

**Table 3:** Univariate and multivariate analysis of overall survival in patients with hilar cholangiocarcinoma

Variables	Univariate analysis		Multivariate analysis	
	HR (95%CI)	P value	HR (95%CI)	P value
Gender	1.107 (0.847-1.447)	0.445		
Male				
Female				
Age(years)	1.131 (0.840-1.522)	0.417		
<65				
>65				
Albumin level(g/L)	0.807 (0.600-1.085)	0.156		
<35				
>35				
RDW	2.267 (1.722-2.984)	< 0.001	1.755 (1.311-2.349)	< 0.001
< 14.95				
> 14.95				
Histologic grade	2.213 (1.631-3.004)	< 0.001	1.591 (1.160-2.183)	0.004
Well/moderate				
poor				
Bismuth type	1.054 (0.912-1.219)	0.473		
Type I, II				
Type III, IV				
Perineural invasion	1.118 (0.856-1.461)	0.413		
Present				
Absent				
T stage	2.301 (1.698-3.118)	< 0.001	0.808 (0.268-2.433)	0.704
T 1,2				
T 3,4				
N stage	4.914 (3.633-6.648)	< 0.001	2.731 (1.564-4.770)	< 0.001
N0				
N1				
AJCC stage	4.845 (3.616-6.490)	< 0.001	2.082 (1.109-3.912)	0.023
I, II				
III, IV				
Tumor diameter(mm)	1.267 (0.961-1.671)	0.093		
≤30				
>30				
Portal vein invasion	2.437 (1.755-3.385)	< 0.001	2.138 (0.928-4.927)	0.074
Present				
Absent				
Hepatic artery invasion	2.027 (1.288-3.189)	0.002	1.395 (0.653-2.980)	0.391
Present				
Absent				

HR, hazard ratio; CI, confidence interval; RDW, red blood cell distribution width.

## DISCUSSION

The current study indicated that RDW is associated with patients’ survival and is an independent risk factor for prognosis in HC.

The RDW is a measure of variability of red blood cell volume, that is routinely examined with the complete blood count and it represents quantitative measure of anisocytosis. An elevated RDW in peripheral blood may be associated with different types of anemias, as well as certain liver disorders and systemic inflammation [14]. Additionally, elevated RDW has also been reported as a predictor of adverse outcomes in patients with myocardial infarction, venous thromboembolism, ischemic stroke and cerebral infarction over a past few years [14–17]. However, recent studies showed that RDW increased in patients with malignant tumors. Some studies indicated that the RDW was dramatically higher in patients with malignances than in healthy persons [18]. Some researches revealed that the RDW was significantly higher in patients with malignant tumors than in those with benign tumors [19, 20], and some reports confirmed that a high RDW was strongly associated with cancer stage and prognosis [21].

The specific mechanism of an elevated RDW in the blood of HC patients is still unknown. The possible mechanisms might involve two aspects: systemic inflammatory response and malnutrition.

Increased RDW values have been reported to be correlated with erythrocyte sedimentation rate (ESR) and high-sensitivity C-reactive protein (CRP) concentration, indicating that the RDW reflects the inflammatory state of the patient [22]. Furthermore, elevated interleukin-6 (IL-6) has been observed in almost all types of tumors acting as a major pro-inflammatory mediator in tumor microenvironment [23]. IL-6 inhibits erythropoietin (EPO) production and downregulates the EPO receptor, ultimately impairing efficient erythropoiesis and causing anisocytosis [24]. Forhecz et al investigated the mechanism of the RDW increase induced by inflammatory reactions and found that inflammatory factors affected iron metabolism, shortened the life of red blood cells, and resulted in the release of large numbers of immature red blood cells from the bone marrow into the peripheral blood circulation in advance; alternatively, inflammatory factors increased the rate of ineffective hematopoiesis in the bone marrow, increased the red blood cell volume heterogeneity in peripheral blood, induced an increase in the RDW, suppressed the stimulating effect of erythropoietin on bone marrow erythroid stem cells, along with preventing the antiapoptotic effect and inhibitory effect of erythropoietin on red blood cell maturation [25]. A growing body of evidence has suggested that systemic inflammatory response plays an important role in cancer progression. Tumor-related inflammatory microenvironment could facilitate tumor growth and metastasis by sustaining proliferation, inhibiting apoptosis, inducing epithelial-to-mesenchymal transition (EMT), initiating angiogenesis, and suppressing host-anti-tumor immunity [26]. As shown in Figure 2, higher WBC and higher NLR were associated with higher RDW. This result might support the idea that high levels of RDW reflect the chronic inflammation status of patients with HC.

Malnutrition owing to direct effect of cancer causing loss of appetite and weight can lead to deficiency of various minerals as well as vitamins such as iron, folate and vitamin B12. It is a well-known fact that RDW is affected by deficiencies of these minerals and vitamins [27]. This is in agreement with our results, lower ALB and lower HGB were associated with higher RDW.

In this study, the cutoff value of RDW is 14.95. It is different from other malignant tumors including lung cancer, prostate cancer esophageal cancer and hepatocellular carcinoma. We speculated that the prognosis-related RDW values may vary for different malignancies. However, we believe that the difference in laboratory measures and the number of patients included may affect the cutoff value of RDW. We hope that through multicenter cooperation, we can incorporate more patients, unify the RDW measurement standards, and achieve a more convincing cutoff value of RDW.

Some limitations of the study should also be taken into account when interpreting the results. Firstly, our study was retrospective with inherent limitations in its design. Thus, some clinical bias was inevitable. We also did not collect relevant laboratory data regarding inflammatory factors (such as the ESR, CRP and ILs), EPO, folic acid, vitamin B12 and serum ferritin concentration. These indicators might greatly help to further elucidation of the mechanism of RDW elevation in HC patients (Figure 2).

In conclusion, RDW is a simple, inexpensive, routinely measured and automatically reported blood test parameter, which reflects the degree of anisocytosis of red blood cells in peripheral blood. Preoperative elevated RDW in the peripheral blood may be regarded as an indicator of systemic inflammatory response which might facilitate HC growth and metastasis. Current evidence suggests that RDW may have clinical significance in predicting OS after surgery in HC patients.

## MATERIALS AND METHODS

### Patients selection

A total of 292 consecutive patients who underwent radical resection for a pathological diagnosis of HC at the West China hospital between January 2005 and February 2012, were retrospectively enrolled and reviewed. The inclusion criteria were as follows: (1) HC was confirmed by pathology examination; (2) patients underwent radical resection (R0 resection). The exclusion criteria included: (1) patients with gallbladder or intrahepatic cholangiocarcinoma extending to the hilum; (2) recurrent or metastatic tumor; (3) non-radical resection (R1 and R2 resection); (4) hematological disorders, autoimmune diseases, systemic inflammatory diseases, renal disease and other cancer; (5) medical treatment with anti-inflammation and (6) patients with preoperative neoadjuvant (chemotherapy and/or radiation) therapy.

### Preoperative workup

Preoperative assessment consisted of medical history, physical examination, laboratory tests and radiography. All patients were evaluated by contrast-enhanced ultrasound, contrast-enhanced computed tomography or magnetic resonance cholangiography along with magnetic resonance cholangiopancreatography to determine the location and extent of the tumor. Biliary drainage, including endoscopic retrograde cholangiopancreatography (ENBD) and percutaneous transhepatic cholangiodrainage (PTCD), was applied in patients having obstructive jaundice with >85 μmol/L total bilirubin. Preoperative portal vein embolization (PVE) was performed in patients with a future remnant liver (FRL) volume <40%.

### Surgical characteristics of the patients

To achieve negative resection margins, different types of surgical approaches, such as segmental bile duct resection, extrahepatic bile duct resection and en bloc resection of the caudate lobe combined with hepatectomy, were adopted. In addition, standard regional lymph node dissection was performed. The surgery was abandoned if metastases to the distant lymph nodes were diagnosed during surgery. According to American Joint Committee on Cancer (AJCC, 7^th^ edition), the locations of regional lymph nodes are defined as follows: along the common bile duct, cystic duct, portal vein and proper hepatic artery [28]. Vascular resection and reconstruction were performed only when vessels could not be detached from the tumor.

### Pathological examination

The pathological evidence of cancer was determined by paraffin sections. All HC included were histopathologically confirmed by experienced pathologists. An R0 resection was defined as the presence of a macroscopically and microscopically tumor-free resection margin. An R1 resection was defined as microscopic evidence of tumor tissue at the resection margin. An R2 resection was defined as macroscopic evidence of tumor tissue at the resection margin.

### Follow up

Whether or not chemotherapy and radiotherapy can benefit HC patients remains controversial. Patients with R0 resection were not treated with postoperative chemotherapy or radiotherapy. All enrolled patients had routine follow-up every three months in the first year and subsequently every six months for at least five years post-surgery. The tumor markers (i.e., serum levels of carbohydrate antigen 19-9 and carcinoembryonic antigen), liver function and ultrasonography were conducted. If there were suspicions of tumor recurrence, contrast-enhanced computed tomography or magnetic resonance imaging were further performed. Tumor recurrence was diagnosed on the basis of the combined findings of typical radiological appearance, quantification of CA19-9 levels, and clinical presentation. The date of the first suspicious radiological finding was recorded as the date of initial disease recurrence.

### Statistical analysis

Patients data were retrospectively collected and statistical analyses were performed using SPSS version 20.0 (SPSS Inc. Chicago, IL, USA). The quantitative variables were expressed as mean (SD) if they presented a normal distribution or otherwise as median and range. Qualitative variables were presented in absolute numbers and percentages. Normally distributed continuous data were compared by means of the Student t test, skewed-distributed by the Mann–Whitney U-test and ordinal data were compared in a χ2 test or Fisher’s exact test. The optimal cutoff value of RDW was determined by the receiver operating characteristic curve (ROC). Survival was described using the Kaplan–Meier method and differences between subgroups were reviewed with the log-rank test. The multivariate analysis for prognostic factors used a Cox proportional hazards model to analyze variables with P < 0.05 in the univariate analyses. Two-sided P values < 0.05 were considered to be statistically significant.

## Abbreviations

RDW: red blood cell distribution width
HC: hilar cholangiocarcinoma
ENBD: endoscopic retrograde cholangiopancreatography
PTCD: percutaneous transhepatic cholangiodrainage
ROC: receiver operating characteristic curve
AJCC: American Joint Committee on Cancer
ALB: albumin
HGB: hemoglobin
WBC: white blood cell
NLR: neutrophil-to-lymphocyte ratio
IL-6: interleukin-6
EPO: erythropoietin
OS: overall survival

## References

[1] DeOliveira ML, Cunningham SC, Cameron JL, Kamangar F, Winter JM, Lillemoe KD, Choti MA, Yeo CJ, Schulick RD (2007). Cholangiocarcinoma: thirty-one-year experience with 564 patients at a single institution. Ann Surg.

[2] Xiong J, Nunes QM, Huang W, Wei A, Ke N, Mai G, Liu X, Hu W (2015). Major hepatectomy in Bismuth types I and II hilar cholangiocarcinoma. J Surg Res.

[3] Rizvi S, Gores GJ (2013). Pathogenesis, diagnosis, and management of cholangiocarcinoma. Gastroenterology.

[4] Tyson GL, El-Serag HB (2011). Risk factors for cholangiocarcinoma. Hepatology.

[5] Molina V, Sampson J, Ferrer J, Díaz A, Ayuso JR, Sánchez-Cabús S, Fuster J, García-Valdecasas JC (2017). Surgical treatment of perihilar cholangiocarcinoma: early results of en bloc portal vein resection. Langenbecks Arch Surg.

[6] Nagino M, Ebata T, Yokoyama Y, Igami T, Sugawara G, Takahashi Y, Nimura Y (2013). Evolution of surgical treatment for perihilar cholangiocarcinoma: a single center 34-year review of 574 consecutive resections. Ann Surg.

[7] Robles R, Sánchez-Bueno F, Ramírez P, Brusadin R, Parrilla P (2013). Liver transplantation for hilar cholangiocarcinoma. World J Gastroenterol.

[8] Yeşil A, Senateş E, Bayoğlu IV, Erdem ED, Demirtunç R, Kurdaş Övünç AO (2011). Red cell distributionwidth: a novel marker of activity in inflammatory bowel disease. Gut and Liver.

[9] Koma Y, Onishi A, Matsuoka H, Oda N, Yokota N, Matsumoto Y, Koyama M, Okada N, Nakashima N, Masuya D, Yoshimatsu H, Suzuki Y (2013). Increased red blood cell distribution width associates with cancer stage and prognosis in patients with lung cancer. PLoS One.

[10] Albayrak S, Zengin K, Tanik S, Bakirtas H, Imamoglu A, Gurdal M (2014). Red cell distribution width: a novel marker of activity in inflammatory bowel disease. Asian Pac J Cancer Prev.

[11] Chen GP, Huang Y, Yang X, Feng JF (2015). A Nomogram to Predict Prognostic Value of Red Cell Distribution Width in Patients with Esophageal Cancer. Mediators Inflamm.

[12] Goyal H, Lippi G, Gjymishka A, John B, Chhabra R, May E (2017). Prognostic significance of red blood cell distribution width in gastrointestinal disorders. World J Gastroenterol.

[13] Beyazit Y, Kekilli M, Ibis M, Kurt M, Sayilir A, Onal IK, Purnak T, Oztas E, Tas A, Yesil Y, Arhan M (2012). Can red cell distribution width help to discriminate benign from malignant biliary obstruction? A retrospective single center analysis. Hepatogastroenterology.

[14] Kust D, Lucijanic M, Urch K, Samija I, Celap I, Kruljac I, Prpic M, Lucijanic I, Matesa N, Bolanca A (2017). Clinical and prognostic significance of anisocytosis measured as a red cell distribution width in patients with colorectal cancer. QJM.

[15] Ellingsen TS, Lappegård J, Skjelbakken T, Brækkan SK, Hansen JB (2015). Red cell distribution width is associated with incident venous thromboembolism (VTE) and case-fatality after VTE in a general population. Thromb Haemost.

[16] Zoller B, Melander O, Svensson P, Engstrom G (2014). Red cell distribution width and risk for venous thromboembolism: a population-based cohort study. Thromb Res.

[17] Ramírez-Moreno JM, Gonzalez-Gomez M, Ollero-Ortiz A, Roa-Montero AM, Gómez-Baquero MJ, Constantino-Silva AB (2013). Relation between red blood cell distribution width and ischemic stroke: a case-control study. Int J Stroke.

[18] Wei TT, Tang QQ, Qin BD, Ma N, Wang LL, Zhou L, Zhong RQ (2016). Elevated red blood cell distribution width is associated with liver function tests in patients with primary hepatocellular carcinoma. Clin Hemorheol Microcirc.

[19] Ay S, Eryilmaz MA, Aksoy N, Okus A, Unlu Y, Sevinc B (2015). Is early detection of colon cancer possible with red blood cell distribution width?. Asian Pac J Cancer Prev.

[20] Seretis C, Seretis F, Lagoudianakis E, Gemenetzis G, Salemis NS (2013). Is red cell distribution width a novel biomarker of breast cancer activity? Data from a pilot study. J Clin Med Res.

[21] Koma Y, Onishi A, Matsuoka H, Oda N, Yokota N, Matsumoto Y, Koyama M, Okada N, Nakashima N, Masuya D, Yoshimatsu H, Suzuki Y (2013). Increased red blood cell distribution width associates with cancer stage and prognosis in patients with lung cancer. PLoS One.

[22] Lippi G, Targher G, Montagnana M, Salvagno GL, Zoppini G, Guidi GC (2009). Relation between red blood cell distribution width and inflammatory biomarkers in a large cohort of unselected outpatients. Arch Pathol Lab Med.

[23] Kumari N, Dwarakanath BS, Das A, Bhatt AN (2016). Role of interleukin-6 in cancer progression and therapeutic resistance. Tumour Biol.

[24] Goyal H, Hu ZD (2017). Prognostic value of red blood cell distribution width in hepatocellular carcinoma. Ann Transl Med.

[25] Förhécz Z, Gombos T, Borgulya G, Pozsonyi Z, Prohászka Z, Jánoskuti L (2009). Red cell distribution width in heart failure: Prediction of clinical events and relationship with markers of ineffective erythropoiesis, inflammation, renal function, and nutritional state. J Am Heart Assoc.

[26] Li Z, Hong N, Robertson M, Wang C, Jiang G (2017). Preoperative red cell distribution width and neutrophil-to-lymphocyte ratio predict survival in patients with epithelial ovarian cancer. Sci Rep.

[27] Goyal H, Gupta S, Singla U (2016). Level of red cell distribution width is affected by various factors. Clin Chem Lab Med.

[28] Groot Koerkamp B, Wiggers JK, Allen PJ, Busch OR, D'Angelica MI, DeMatteo RP, Fong Y, Gonen M, Gouma DJ, Kingham TP, van Gulik TM, Jarnagin WR (2014). American Joint Committee on Cancer staging for resected perihilar cholangiocarcinoma: a comparison of the 6th and 7th editions. HPB (Oxford).

